# Harmful Waste Products as Novel Immune Modulators for Treating Inflammatory Arthritis?

**DOI:** 10.1371/journal.pmed.0030385

**Published:** 2006-09-12

**Authors:** Andrew P Cope

## Abstract

Cope discusses a new study in rats suggesting that oxidative burst inducers might have a role to play in treating inflammatory arthritis.

## The Role of Reactive Oxygen Species

For many years, reactive oxygen species (ROS), including superoxide, hydrogen peroxide, and hydroxyl radicals, and their reaction products, were classically described as harmful by-products of aerobic metabolism capable of causing DNA mutations, lipid peroxidation, and protein oxidation [1]. The identification of enzymes such as superoxide dismutase, catalase, and peroxidase that served to eliminate these waste products rather substantiated this view. It soon became clear, however, that there existed a family of enzymes whose function it was to deliberately generate ROS. The first of these was NADPH oxidase, which is responsible for the respiratory burst in neutrophils and macrophages in response to microbes or inflammatory cytokines [2]. The catalytic centre of NADPH oxidase is the membrane-associated protein gp91^phox^ (see Glossary) complexed with p22^phox^. Activation requires association with a phosphorylated form of p47^phox^ (also known as Ncf1), p67^phox^, and the small GTPase Rac (Figure 1). p47^phox^ deficiency in humans leads to neutrophil dysfunction and chronic granulomatous disease (CGD), a primary immunodeficiency disorder characterised by the inability to eradicate bacterial infections [3]. Since the late 1990s, six human gp91phox homologues have been identified, each with distinct functions [1].

**Figure 1:**
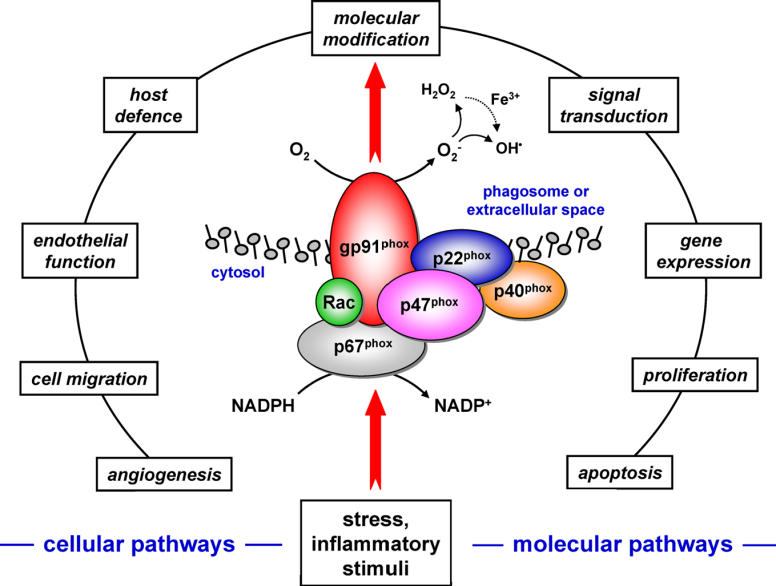
Structure and Function of the NADPH Oxidase Complex Schematic of the molecular composition of the NADPH oxidase complex. The principal subunits are shown, together with some of the key cellular and molecular modifications that arise following activation of stress pathways and the generation of superoxide (O_2_^–^). Two molecules of superoxide can react to generate hydrogen peroxide (H_2_O_2_). In the presence of iron, superoxide and H_2_O_2_ react to generate hydroxyl radicals (OH•). Through their effects on protein modification and lipid peroxidation, reactive oxygen species exert pleiotropic effects on multiple molecular and cellular pathways.

Besides a role in phagocyte function and host defence, a large amount of evidence points to important roles for ROS in cell proliferation, apoptosis, angiogenesis, endocrine-related functions, and oxidative modification of the extracellular matrix. Indeed, increased ROS have been documented at sites of inflammation, such as synovial joints of patients with inflammatory arthritis (e.g., rheumatoid arthritis [RA]), and circulating neutrophils and monocytes from patients with RA have increased NADPH oxidase activity [4,5].

Interestingly, polyarthritis has also been described in patients with CGD. More recently, induction of arthritis in mice deficient for the p47^phox^ subunit of NADPH oxidase was shown to induce granulomatous synovitis and exaggerated matrix destruction associated with enhanced expression of inflammatory mediators [6]. Although the mechanism for this paradoxical relationship between deficient NADPH oxidase activity and inflammatory arthritis has not been elaborated until now, these findings certainly imply a more complex role for the NADPH oxidase complex in chronic inflammation than previously thought.

## Oxidative Burst Capacity and Inflammatory Arthritis

Chronic inflammatory syndromes such as RA are complex polygenic diseases in which it is proposed that inheritance of gene polymorphism predisposes to distinct phenotypes such as the magnitude of the inflammatory response, the development of autoantibodies, or destruction of cartilage and bone. One productive approach for screening for such gene polymorphism has been to define in relevant rodent models chromosomal segments associated with each disease phenotype. Multiple backcrossing of these chromosomal segments to the disease-prone strain generates congenic animals, which are invaluable tools for further study. For example, linkage analysis in rats susceptible or resistant to pristane-induced arthritis (PIA, an inflammatory arthritis induced with the alkane oil pristane) has defined quantitative trait loci (QTL) that associate with disease severity and cartilage destruction.

In 2003, Holmdahl and colleagues reported the results of a detailed analysis of one such QTL, *pia4*, which is implicated not only with arthritis, but also in rodent models of multiple sclerosis and uveitis [7]. Introduction of the 20cM *pia4* fragment from the arthritis-resistant E3 strain into arthritis-prone DA rats by serial backcrosses substantially reduced arthritis severity. By positional cloning, two structural polymorphisms of the *Ncf1* gene (encoding p47^phox^) were identified in arthritis-susceptible DA rats characterised at the amino acid level by M106V and M153T substitutions. While the allelic variant had no effect on *Ncf1* gene expression, the disease-associated allele conferred an unexpected reduction in oxidative burst upon stimulation of rat peritoneal cells with phorbol ester, presumably through the effects of the mutations on the function of the NADPH oxidase complex. Strikingly, this defective respiratory burst could be reversed in vivo by treating arthritis-prone DA rats with phytol, an alkane similar to pristane but that induces a robust reactive oxygen burst. Treatment not only restored the respiratory burst but also protected rats from developing arthritis.

## A New Rodent Study

In a new rodent study in *PLoS Medicine*, Holmdahl and colleagues explore in more detail the relationship between oxidative burst capacity and predisposition to inflammatory arthritis in an approach that has implications for better understanding this paradoxical relationship in humans [8]. First, they compared how alkane derivatives such as pristane and phytol function in terms of oxidative burst capacity and arthritogenicity, and discovered that arthritis induction was independent of oxidative burst. Thus, while both phytol and pristane were potent inducers of oxidative burst in granulocytes in vitro, pristane induced, whereas phytol protected rats from disease. Importantly, phytol treatment restored the oxidative burst of Ncf1^DA^ rats to levels observed in splenic granulocytes carrying the Ncf1^E3^ protective allele, whilst phytol induced only very modest increases in ROS production in T cells.

**Glossary**

**Adoptive transfer:** Transfer of cells from donor to recipient to induce or modify disease

**gp91^phox^:** 91-kD glycoprotein subunit of NADPH oxidase, a phagocyte oxidase (hence, “phox”)

**LAT:** Linker for activation of T cells, an adaptor protein involved in transmitting receptor proximal signals from the TCR to downstream intracellular signal transduction pathways

**Multiple backcrossing:** Sequential breeding of F1 offspring with an animal derived from a particular inbred strain of mouse or rat; usually used to introduce a genetic fragment from one strain to another

**p22^phox^:** 22-kD subunit of NADPH oxidase

**p47^phox^ (Ncf1):** 47-kD subunit of NADPH oxidase (also known as Ncf1)

**p67^phox^:** 67-kD subunit of NADPH oxidase (also known as Ncf2)

**Positional cloning:** Sequencing genes in the vicinity of phenotype-linked microsatellites (in this case, *Pia4*–linked) to identify the polymorphisms associated with the particular characteristic of interest (in this case, altered severity of arthritis)

**Pristane-induced arthritis:** Induction of arthritis using the alkane oil pristane, commonly used as a model of RA

**Rac:** A small GTP-binding protein involved in cell signalling

**TCR ζ** An invariant subunit of the T cell antigen receptor complex, also known as CD3ζ

**Thiols:** Sulphhydryl groups (e.g., the –SH group is a reduced thiol)

Second, the authors explored the effects of phytol in different arthritis models, including collagen-induced arthritis, anti-collagen II antibody-induced arthritis, and non-oil collagen-induced arthritis, demonstrating both protective and therapeutic effects in all models; similar effects were observed in rats with normal oxidative burst capacity. Treatment resulted in reduction of the inflammatory response, attenuation of cell-mediated immunity, and downregulation of markers of cartilage destruction, modifying clinical disease to an extent at least as good as methotrexate or TNF blockade (now considered gold-standard therapy for RA). Histological analysis of joint sections confirmed the joint-protecting effects of phytol.

Finally, an elegant series of experiments involving the transfer of T cells from pristane-inoculated rats to unmanipulated donor animals demonstrated, among other things, that the effects of phytol on arthritogenic T cells were rapid, since treatment of the donor T cells with phytol for as little as three hours was sufficient to prevent the development of arthritis in recipient rats. By contrast, adoptive transfer of a mixture of T cells from control and phytol-treated animals failed to inhibit arthritis, suggesting that the disease-modifying effects of phytol on T cells are cell intrinsic, affecting only those cells directly exposed to the therapeutic agent.

## Study Implications

Why should enhancing oxidative burst and ROS production paradoxically ameliorate arthritis? There are several possible explanations, none of which would necessarily be mutually exclusive. We know, for example, that deficiency of *Ncf1* in mice and humans predisposes to repeated insult with infectious pathogens [3,9]. Accordingly, chronic subclinical infection in Ncf1^DA^ rats could explain predisposition to disease through persistent activation of innate immune responses. However, the authors argue that arthritis occurs in both conventional and specific pathogen-free facilities with equivalent severity, implying that the impact of deficient versus mutant *Ncf1* gene products on host immunity might be distinct. Detailed analysis of the response of *Ncf1* congenic rat strains to challenge with bacterial pathogens should now be undertaken to rule this out.

Another conundrum arising from this work is the strikingly different levels of ROS induced in granulocytes and T cells after treatment with phytol in vitro; effects on T cell ROS levels were modest at best. So how could phytol regulate T cell reactivity? There is literature pointing to indirect effects of granulocyte- or macrophage-derived ROS on T cell phenotype and function. For example, coculture of T cells with ROS-producing neutrophils reduces T-cell reactivity as well as cytokine and proliferative responses [10]. At the molecular level, several mechanisms might be implicated, including alterations of expression or function of key T cell receptor (TCR)–signalling molecules including the TCR chain and the transmembrane adaptor protein linker for activation of T cells (LAT) [10–12]. The oxidation status of T cells exposed to extracellular ROS might also influence the function of phosphatases that are known to be exquisitely sensitive to the oxidation of cysteine in the catalytic site [13]. This in turn might alter thresholds of T-cell reactivity. Increased susceptibility to apoptosis associated with exaggerated oxidative burst has also been reported, but this does not appear to be playing a major role in Holmdahl and colleagues' rat models. Finally, we should not exclude the possibility that Ncf1 could play a role in as-yet undefined NADPH oxidase–independent pathways, perhaps exerting a negative regulatory role on intracellular signals involved in immune homeostasis.

One intriguing possibility may relate to recent findings suggesting that cell surface thiols (–SH) are targets of redox regulation [14], which could directly influence thresholds of T-cell reactivity [15]. According to this model, a “normal” oxidising extracellular environment would favour oxidation of cell surface molecules and receptors, thereby maintaining T cells in check. Under conditions that predispose to a defective oxidative burst (which would include inheritance of *Ncf1* mutations), increased surface thiols would lower thresholds of T cell activation, permitting uncontrolled expansion of effector T cells in vivo. This would presumably influence thymic development as well, since lowering TCR-signalling thresholds selects a repertoire of T cells that might otherwise die by neglect when failing to receive appropriate survival signals.

These intriguing results are important because they illustrate another means whereby redox reactions might be manipulated in the clinic. This would be especially attractive if, as this study demonstrates, the effects are rapid and reversible, because robust immunological and molecular tools are already available to monitor such interventions. This approach would be all the more appealing if genetic variation of subunits of the NADPH oxidase complex is associated with susceptibility to autoimmunity in humans. While the data provide unambiguous evidence for a dual role for the oxidative burst in innate and adaptive immune responses, it is now abundantly clear that waste products are not all bad.

## Abbreviations

RA: rheumatoid arthritis
ROS: reactive oxygen species
TCR: T cell receptor
